# The reliability of a 10-test package for patients with prolonged back and neck pain: could an examiner without formal medical education be used without loss of quality? A methodological study

**DOI:** 10.1186/1471-2474-8-31

**Published:** 2007-04-03

**Authors:** Odd Lindell, Lars Eriksson, Lars-Erik Strender

**Affiliations:** 1 Center for Family Medicine, Karolinska Institutet, Alfred Nobels allé 12, SE-141 83 Huddinge, Sweden; 2 Rehab Station Stockholm, Frösundaviks allé 13, SE-169 89 Solna, Sweden; 3 Haninge FysioCenter, Rörvägen 4, SE-136 50 Haninge, Sweden

## Abstract

**Background:**

In the rehabilitation of patients with prolonged back and neck pain, the physical impairment should be assessed. Previous research has exclusively engaged medically educated examiners, mostly physiotherapists. However, less biased evaluations of efforts at rehabilitation might be achieved by personnel standing outside the treatment work itself. Therefore, if medically untrained examiners could be used without cost to the quality, this might produce a better evaluation at defensible cost and could also be useful in a research context. The aim of this study was to answer the question: given a 10-test package for patients with prolonged back and neck pain, could an examiner without formal medical education be used without loss of quality? Five of the ten tests required the examiner to keep a firm hold against the foundation of those parts of the participant's body that were not supposed to move during the test.

**Methods:**

Examination by an experienced physiotherapist (A) in performing the package was compared with that by a research assistant (B) without formal medical education. The reliability, including inter- and intra-rater reliability, was assessed. In the inter-rater reliability study, 50 participants (30 patients + 20 healthy subjects) were tested once each by A and B. In the intra-rater reliability study, the 20 healthy subjects were tested twice by A or B. One-way ANOVA intra-class-correlation coefficient (ICC) was calculated and its possible systematic error was determined using a *t*-test.

**Results:**

All five tests that required no manual fixation had acceptable reliability (ICC > .60 and no indication of systematic error). Only one of the five tests that required fixation had acceptable reliability. The difference (five vs. one) was significant (*p *= .01).

**Conclusion:**

In a 10-test package for patients with prolonged back and neck pain, an examiner without formal medical education could be used without loss of quality, at least for the five tests requiring no manual fixation. To make our results more generalizable and their implications more searching, a similar study should be conducted with two or more examiners with and without formal medical education, and the intra-rater reliability study should also include patients and involve more participants.

## Background

In the industrial world, back and neck pain, i.e. pain in the lumbar, thoracic and/or cervical spine, constitutes the largest diagnostic group underlying sick-listing, including disability pensions [1]. In the rehabilitation of patients with prolonged back and neck pain, it is necessary to assess the physical impairment, i.e. the pathological, anatomical or physiological abnormality of structure or function leading to loss of normal ability [2]. The vast majority (around 95%) of these patients suffer from non-specific back and neck pain and require no specific surgical, rheumatological or neurological treatment. Therefore, the focus of assessment of prolonged back and neck pain is on abnormality of function [3]. Acceptable reliability of an assessment method includes acceptable inter- and intra-rater reliability, i.e. it requires that the measurements are comparable when performed (a) on the same subject by numerous examiners and (b) on several occasions by the same examiner [4]. Besides reliability, validity, i.e. the degree to which a useful interpretation can be inferred from a measurement [5], is an important aspect of an assessment method. For example, if in a lifting test the patient is able to lift 10 kg as maximum, how is the clinical meaning for that individual interpreted? However, the concept of validity is outside the framework of this study.

Forward bending, when it is measured as the distance of the fingertip to the floor and by the modified Schober test, had acceptable reliability [6], as did lateral bending measured as the distance moved by the hand down the outside of the thigh [7]. Trunk rotation and active-straight-leg raise have been examined by goniometers, but those tests were not validated [8]. Cervical bending and rotation as investigated by the CROM instrument demonstrated acceptable reliability [9,10]. Isometric endurance of the abdominal muscles as examined in the form of a partial sit-up had acceptable reliability [11]. Moreau et al. [12] found that the Biering-Sørensen test was the most useful of the isometric back-extension endurance tests. In an 11-test package, six of the tests had acceptable reliability [4]; in an 8-test package, only one test had acceptable reliability [13].

Patients with prolonged back and neck pain were offered rehabilitation at a Swedish primary-care centre. The physiotherapists at the centre used a 10-test package. Most of the tests in this package had been validated in previous studies by comparing the results obtained by medically trained examiners. From August 2000 to January 2006 a randomized controlled trial was running, in which rehabilitation at the centre was compared with traditional primary care. At the time of inclusion and one year later, each patient in the randomized controlled trial met a research assistant at that patient's health centre. Among other items, the patients performed the 10-test package. For practical and economic reasons it was appropriate for the person who administrated the study and visited the different health centres also to execute the test package. Although the research assistant had no formal medical education, this seemed reasonable, since the tests were standardized and easy to perform. In some reliability studies, chiropractors [14], naprapaths [15] or physicians [6,15-18] have been represented. The vast majority of reliability studies, however, have been performed with physiotherapists as examiners [4,9-11,13,19-21]. We have found no study of reliability in which examiners without formal medical education were engaged. However, the evaluation of rehabilitation efforts might be less biased if performed by personnel standing outside the treatment work itself. It seems economically unrealistic for ordinary clinics to keep medically-trained personnel only for assessment tasks. Therefore, if medically untrained examiners could be used without decreased quality, this might produce a better assessment of outcome at defensible cost and could also be useful in a research context.

The aim of this study was to answer the question: given a 10-test package for patients with prolonged back and neck pain, could an examiner without formal medical education be used without loss of quality?

## Methods

### Settings

The study was performed in Haninge, a rural district 25 km south-east of Stockholm, at a primary-care rehabilitation centre and a physiotherapy centre situated next door.

### Examiners

In appraising the assessment work of a medically untrained examiner it seemed logical to use an experienced physiotherapist as the gold standard.

Examiner A (LE) had the highest Swedish degree in orthopaedic manual therapy and had been working as a physiotherapist for ten years. Examiner B had a B.A. (Batchelor of Arts) in psychology but no formal medical education. She had been working as a research assistant with purely administrative tasks for 2 1/2 years and had no previous vocational experience of manual contact with patients. B was prepared for this reliability study by (a) four hours' training in the performance of the 10-test package and (b) practising the package during the autumn of 2000 on barely 40 patients who were included in the above-mentioned randomized controlled trial.

### Subjects

Fifty participants were included and gave their consent to participate in the study: 30 patients with prolonged back and/or neck pain, and 20 healthy subjects.

#### Patients

From March until September 2001, a total of 30 patients were recruited at the physiotherapy centre. Seventeen were females (mean (*m*) 41.5, range (*r*) 28–60, years) and 13 males (*m *42.4, *r *20–63, years). They were supplied with both verbal and written information.

##### Inclusion criteria

1. Back and/or neck pain for more than four weeks. 2. The patient was considered able to execute the whole 10-test package.

##### Exclusion criteria

1. Such severe pain or dysfunction that it might be harmful for the patient to participate. 2. Whiplash-associated disorders. 3. Inability to read the written information.

Thirty-one consecutive patients fulfilling the criteria were asked to participate in the study. All but one agreed.

#### Healthy subjects

From February until September 2001, 20 healthy subjects were recruited among the staff at the rehabilitation centre and the physiotherapy centre. Fourteen were females (*m *36.2, *r *22–55, years) and six males (*m *40.2, *r *28–53, years). Twenty staff members (physiotherapists, physicians and receptionists) were asked consecutively and all of them agreed to participate.

### The 10-test package

Four tests included motion in one direction only. Four comprised motion to the right and to the left, and one involved motion forward and backward. A lifting test included a lumbar and a cervical sub-test. This resulted in ten tests composed of 16 sub-tests.

Five of the ten tests required that the examiner kept a firm hold against the foundation of those parts of the participant's body that were not supposed to move during the test. This manual fixation was done to eliminate misleading co-movements from those parts.

The package followed the protocol of previous studies, with some modifications. We used the widely-adopted modification of the Schober test by Macrae and Wright [22]. To save examination time, we simplified the procedures for another two original tests, the Biering-Sørensen test and the PILE test (see below). The total examination time of the package was approximately 30 minutes. A detailed description is given below.

#### 1. Forward bending

The participant (P) stood barefoot with the heels together. P bent forward, keeping the knees straight and with the arms straightened out downwards the floor. When P had bent maximally, the examiner (E) measured the distance between the middle-finger tip and the floor, to within 1 cm, with a wooden stick. If the floor was reached, the distance was noted as 0 cm [6].

#### 2. Modified Schober

P stood with the feet together. Three dots were marked: dot a between the lowest lumbar spinal process and sacrum, dot b 10 cm above and dot c 5 cm beneath a. P bent forward, keeping the knees straight. The distance b-c when P was bent maximally forward was measured with a tape to within 1 cm. The difference of b-c when maximally bent forward and standing was noted. Normally, b-c increases by at least 5 cm [22].

#### 3. Lateral bending (right/left)

P stood with 20 cm between the feet and with the back, neck, back of the head and shoulders against a wall and the arms loosely against the sides of the body. The middle-finger tip positions on the outside of the thighs were marked with dot a. P bent to the right side, keeping the knees straight and without losing contact between the shoulders and the wall. In the maximally bent position, the middle-finger tip position on the right thigh was marked by dot b. The same procedure was performed on the left side. The distances a-b on the right and left thighs were measured with a tape to within 1 cm [7].

#### 4. Trunk rotation (right/left)

P sat on a stool with the knees together holding a rod horizontally in the frontal plane across the upper sternum and the front of the deltoid muscles. From the ends of the rod, a line with a plumb weight hung down pointing at a semicircular protractor lying on the floor under and in front of P. In the initial position, the base line of the protractor was in the same frontal plane as the rod and the middle of the base line was directly below the middle of the rod. E stood behind P holding the lower part of P's body still by firmly pressing the iliac crests down towards the seat of the stool. P rotated the trunk maximally to the right. The maximally rotated position was read, to within 5 degrees, where the plumb weight pointed at the protractor. The same procedure was performed on the left side [8].

#### 5. Active-straight-leg raise (right/left)

P was lying supine on a couch with the knees straight. An MIE meter was placed on the lower part of the right leg at the tuberositas tibiae. While the left leg was held in its initial position by E, P raised the right leg, keeping the knee straight. When the leg was maximally raised, the angle between the leg and the horizontal plane was read to within 1 degree. The same procedure was performed with the right leg fixed to the couch and the left leg raised [8].

#### 6. Cervical bending (forward/backward)

P sat on a chair with the head in a neutral position. A CROM meter was placed on the head. E held P's thoracic and lumbar spine fixed to the back support of the chair. P bent the head forward and then backward. In the maximally bent positions, the angle between the head and the vertical line was read to within 1 degree [9].

#### 7. Cervical rotation (right/left)

The same procedure as in test 6, except that P rotated the head to the right and then to the left. The angle between the head in neutral and in maximally rotated position was read to within 1 degree [9].

#### 8. Abdominal endurance

P was lying supine on a couch with the knees bent at 90°, the soles of the feet on the couch and the palms resting on the front of the thighs. P performed a sit-up, with the fingertips touching the upper part of the patellae, and sustained this position as long as possible. The maximal sit-up time, until the fingers lost contact with the patellae, was measured with a stop-watch to within 1 second [11].

#### 9. Modified Biering-Sørensen

P was lying prone with the lower part of the body, from the upper part of the iliac crest downwards, placed on a couch. The upper part of the body hung down from the short side of the couch, resting on the seat of a chair 2 dm beneath the level of the couch. E held P's feet fixed to the couch. P lifted the upper body from the seat and held it straight out from the edge of the couch, with the arms folded across the chest. The maximal time for which P was able to keep the unsupported upper body horizontal was measured with a stop-watch to within 1 second.

##### Modifications

In the original Biering-Sørensen, the buttocks and legs are fixed by three canvas straps and there is an upper time limit of 240 seconds [6].

#### 10. Modified PILE (lumbar/cervical)

PILE = Progressive Iso-inertial Lifting Evaluation.

##### Modified PILE lumbar

P lifted a tray with weights (plastic bottles filled with sand) from the floor to a 75-cm-high table and back again to the floor. The table was placed 90° to the left of P, which added a twisting factor. An electronic pulse-counter was attached to P's thorax. The starting weight was 4 kg. E added 2 kg after each successful attempt. Each attempt had to be carried out within 20 seconds. The weight managed during the last lifting moment was recorded as the test result. The test was discontinued if the heart rate reached 85% of the estimated maximal heart rate or if the load reached 55% of the body weight.

##### Modified PILE cervical

This sub-test was carried out as described above, except that P stood in front of the table and lifted the tray from the table up to a 50-cm-high platform (i.e. 125 cm above the floor). The platform was placed on the left side of the table, which added a twisting factor.

##### Modifications

In the original PILE, the table is 76 cm high, the platform is 137 cm above the floor, men and women have different weights at the start (3.6 vs. 5.9 kg) and different weights are added to men and women (2.25 vs. 4.5 kg), and the result is adjusted for the body weight [16]. Our modifications are in line with Lindström et al. ([8]; Lindström, personal communication, 2000).

### Examination procedure

The test package was performed at different times of day. Along with the agreement to participate, the participants received identical instructions, both verbally and in written form, from a manual produced for this study. They were to wear training clothes or underclothes, not to do any warming up, and to perform the tests to their maximum capacity within the limits of exertion and pain; they could discontinue whenever they wanted. The participants were also informed that the examiners were a physiotherapist and a research assistant. The patients were not informed about which of the two examiners they were seeing. The healthy subjects could not be blinded to the examiner because they were co-workers of one or both of the examiners. Whether A or B would conduct the first examination was randomized by envelopes, which were prepared by an independent statistician and opened immediately before the first test. Close to the start of the examination the participant was once again verbally instructed to perform the tests to his or her maximum capacity within the limits of exertion and pain, and was reminded that the tests could be discontinued whenever he or she wanted. The test package was then conducted straight through without a break and without further verbal communication, except for purely technical instructions on how to perform the test. Before the first and after the last test of the package, the participants were asked to estimate their exertion on Borg's 20-point scale [23] and their level of pain on Borg's 10-point scale [24].

The participants and the examiners were given no results on any occasion until all the tests were completed. The participants were asked not to tell the second examiner their experiences at the first examination.

The study was approved by the local ethics committee at Karolinska University Hospital, Huddinge, Sweden.

### Inter-rater reliability study

The 30 patients and 20 healthy subjects were first tested by one of the examiners (examination 1). After a break for 30 minutes, they were re-tested by the other examiner (examination 2).

### Intra-rater reliability study

The 20 healthy subjects participated. Examiners A and B tested ten healthy subjects each. After examination 2, the subjects rested for another 30 minutes and were then re-tested (examination 3) by the same examiner as at examination 1.

The reason for including only healthy subjects in the intra-rater reliability study was that we considered three consecutive examinations too much of a strain for the patients to be ethically defensible; it would also have made the results of the third examination difficult to interpret.

In total, the patients and the healthy subjects were occupied in the study for approximately 1 1/2 and 2 1/2 hours respectively.

### Statistics

Alhough the intra-class correlation coefficient (ICC) is questioned by some authors [25], it is the basic measure in most reliability studies involving continuous data (degrees, centimetres, etc.) [10,13,17,20,26,27]. The ICC increases with the degree of reliability up to a maximum of 1.00 for identical ratings [28]. We calculated the one-way ANOVA (analysis of variance) ICC, random-effects model, and its 95% confidence interval (CI) as described by Haas [28]. We also calculated the standard error of measurement (SEM) of the ICC [29]. The 95% CI is a band of values that, with 95% confidence, contains the true reliability. A narrow CI suggests a more precise estimate of reliability. The SEM enables the reliability of a measurement expressed in the units of the measurement of interest, such as degrees or centimetres, to be assessed. As such, it is valuable for the clinician because it provides guidance on whether the measured change is due to measurement error or to real change [27].

There is a lack of consensus concerning the cut-off values for ICC. For example, Rheault et al. [10] considered ICC > .80 to indicate high reliability and ICC > .60 up to and including .80 to represent moderate reliability. Horneij et al. [13] defined an ICC > .75 as excellent reliability and .40–.75 as fair to good reliability. We chose to consider an ICC > .60 to indicate acceptable reliability and an ICC ≤ .60 to indicate poor reliability, which is a modification of Landis and Koch [30] and in line with the recommendation of Chinn [31].

For each sub-test, the mean difference between the measurements and its 95% CI were calculated. The possible systematic error of the ICC was calculated, using a *t*-test to evaluate the mean difference [17]. We considered a sub-test to have acceptable inter- or intra-rater reliability when ICC was > .60 and there was no significant, systematic error. A test was considered to have acceptable reliability when it had (1) acceptable inter-rater reliability for the 50 participants, (2) acceptable intra-rater reliability for both examiners A and B and (3), for tests comprising two sub-tests, when both sub-tests had acceptable inter- and intra-rater reliability. The proportions of tests that showed acceptable inter-rater reliability were calculated for the patients and for the healthy subjects, and for the five tests that required manual fixation and the five that did not. The proportions of tests with acceptable intra-rater reliability were calculated for A and B and for the tests that did and did not require manual fixation. The proportions of tests with acceptable reliability were calculated for the tests that did and did not require manual fixation. The mutual proportions were then compared by a *z*-test [32].

For each sub-test, scatter plots were used to visualize the agreement. The plots were constructed from the difference between the measurements and the mean difference, and the limits of agreement were indicated by the 95% CI of the mean difference [33].

The exertion and pain before and after each examination were analysed. The difference between examinations 1 and 2 of the 50 participants was compared by the Wilcoxon sign-rank test. The differences between examinations 1 and 3 and the differences between the healthy subjects of examiners A and B were compared by the Wilcoxon rank-sum test [34].

A *p*-value < .05 was considered statistically significant. The statistical calculations were performed and the figures constructed using STATA, version 9.1.

## Results

All 50 participants completed all the tests.

### Inter-rater reliability

Seven of the ten tests had acceptable inter-rater reliability (Table 1). Three tests had poor inter-rater reliability: active-straight-leg raise, cervical bending and modified Biering-Sørensen.

For the patients and the healthy subjects, seven and four of the ten tests respectively had acceptable inter-rater reliability (not significant (NS)).

All five tests that required no manual fixation by the examiner had acceptable inter-rater reliability, compared with two of the five tests that required such fixation. The difference in proportion (five vs. two out of five tests) was significant (*p *= .04).

**Figure 1:**
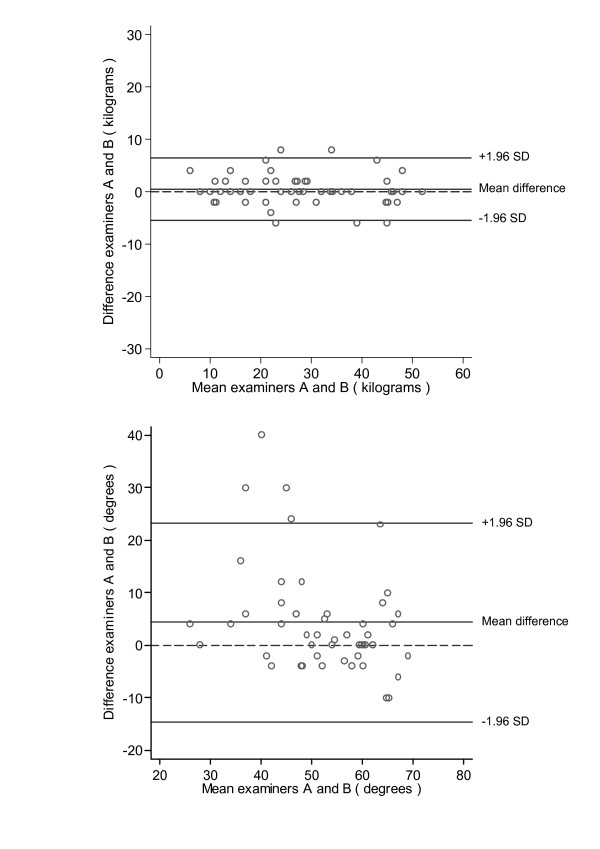
**a-b. Inter-rater reliability. Fifty participants tested by A (the physiotherapist) and B (the research assistant)**. The difference between the measurements by A and B against the mean of the measurements by A and B with 95% limits of agreement (= the mean difference of the measurements with 95% CI). 1 a. Modified PILE lumbar. Acceptable agreement. The mean difference is close to the zero line, which indicates a small systematic error. The limits of agreement are narrow, which indicates a small random error. 1 b. Cervical bending forward. Poor agreement. The mean difference is fairly far from the zero line and the limits of agreement are wide, which indicates high systematic and random error.

Examples of scatter plots showing acceptable and poor agreement, respectively, are shown in Figure 1a–b.

The exertion and the pain before and after examination 1 did not differ significantly from those before and after examination 2 (data not shown).

### Intra-rater reliability

For examiner A (the physiotherapist), all ten tests had acceptable intra-rater reliability (Table 2). For examiner B (the research assistant), eight tests, i.e. all but the trunk rotation and the modified Biering-Sørensen, had acceptable intra-rater reliability (NS).

All the tests requiring no manual fixation had acceptable intra-rater reliability for both A and B. Of the five tests that required manual fixation, five and three tests had acceptable intra-rater reliability for A and B, respectively (NS).

**Figure 2:**
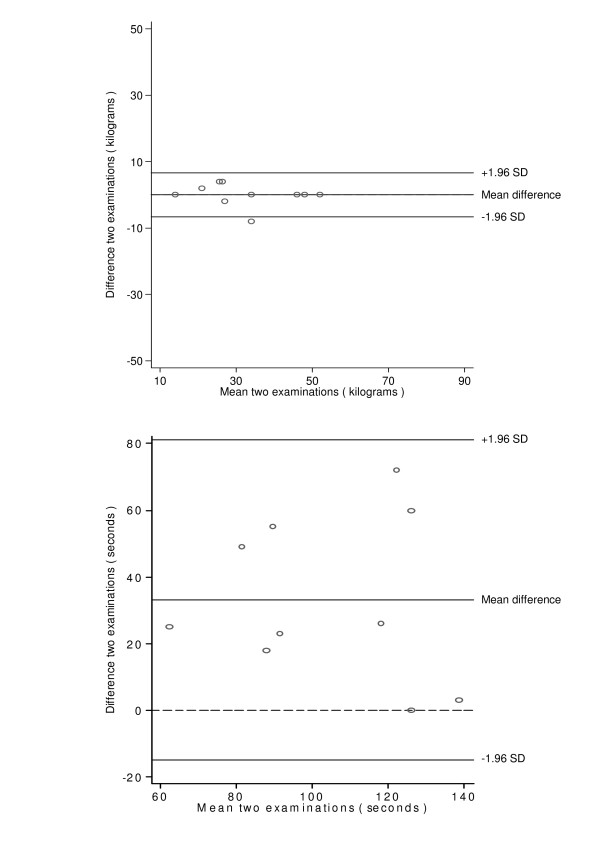
**a-b. Intra-rater reliability. Ten healthy subjects tested twice by B**. The difference between the two examinations against the mean of the two examinations with 95% limits of agreement. Further explanations in Figure 1 a-b. 2 a. Modified PILE lumbar. Acceptable agreement. The mean difference is identical to the zero line, which indicates a very small systematic error. The limits of agreement are narrow, which indicates a small random error. 2 b. Modified Biering-Sørensen. Poor agreement. The mean difference is far from the zero line and the limits of agreement are very wide, which indicates high systematic and random error.

Examples of scatter plots showing acceptable and poor agreement, respectively, are shown in Figure 2a–b.

The exertion and the pain before and after examinations 1 and 3 did not differ significantly between the ten healthy subjects of A and B (data not shown).

### Reliability

All five tests requiring no manual fixation had acceptable reliability, i.e. acceptable inter-rater reliability, acceptable intra-rater reliability for both A and B and, if the test was composed of two sub-tests, acceptable inter- and intra-rater reliability for both sub-tests (Tables 1 and 2). Those tests were forward bending, modified Schober, lateral bending, abdominal endurance and modified PILE.

The five tests that required manual fixation – trunk rotation, active-straight-leg raise, cervical bending, cervical rotation and modified Biering-Sørensen – all had poor reliability except cervical rotation. The difference in proportion (five vs. one out of five tests) was significant (*p *= .01).

## Discussion

The aim of this study was to answer the question: given a 10-test package for patients with prolonged back and neck pain, could an examiner without formal medical education be used without loss of quality?

Was the composition of the 10-test package suitable for answering this question? From our knowledge, there has been no previous reliability study involving a medically untrained examiner. However, numerous studies have elucidated the problem of achieving agreement between medically skilled examiners, including both the choice of tests and the circumstances during which the examinations are performed. Some reliability studies include tests of inter-segmental mobility, i.e. passive mobility between two vertebrae levels [20]. Strender et al. [18] demonstrated the acceptable inter-rater reliability of such tests, provided that the examination situation is ideal. An ideal situation implies that the examiners have been able to standardize their techniques by working together for a sufficiently long period. In non-ideal conditions, Fjellner et al. [21] obtained acceptable inter-rater reliability in several tests of general motion but in few tests of inter-segmental mobility. As the everyday clinical situation is seldom ideal, we chose motion tests for our test package that exclusively concerned general mobility. The comparatively high proportion of tests with acceptable inter-rater reliability in our study (seven out of ten tests) supported this despite the non-ideal conditions. Notwithstanding the absence of previous references, it seems reasonable to predict that an examiner without medical education and practice will experience even greater difficulties in performing a standardized technique of manual fixation than an examiner with such skills. In support of this, the tests in our package that required fixation tended to have a higher proportion of acceptable intra-rater reliability for the physiotherapist than for the research assistant (five vs. three tests), though the difference was not significant. As a matter of fact, all the technically least advanced of our tests, i.e. the five that required no manual fixation by the examiner, had acceptable inter-rater reliability (five out of five tests). The proportion was significantly lower for the five tests requiring manual fixation (two out of five tests). This is consistent with the study of Bertilsson et al. [15], in which a simple sensitivity test had acceptable inter-rater reliability while several more sophisticated tests had not. The abdominal endurance had acceptable reliability, as against the study of Moreland et al. [26], in which the hands of the participant were held on the cheeks. In our study, as in the studies of Hyytiäinen et al. [11] and Lindström et al. [8], the hands were stretched out towards the patellae. The test package was inexpensive and easy to perform. Our study indicates, however, that Biering-Sørensen, when it is simplified as we described, has poor reliability. We found that the modified PILE had acceptable reliability, which complements the study of Lindström et al. [8]. They found this modification to have good validity, i.e. that the lifting capacity, when measured as described, correlated significantly with the rate of return to work, but their study included no test of reliability. Without exception, the five tests requiring no manual fixation had acceptable reliability. Five of the tests required such fixation, including the modified Biering-Sørensen and the previously unvalidated tests of trunk rotation and active-straight-leg raise. Only one of them (cervical rotation) had acceptable reliability. This difference (five vs. one) was significant (*p *= .01). All tests requiring no manual fixation had acceptable intra-rater reliability for both A and B. Concerning the composition of our test package, it seemed right to include motion tests exclusively concerned with general mobility, but we underestimated the technical difficulties of manual fixation. Thus, the composition of the 10-test package proved to be fairly suitable for answering the question of this study, indicating *inter alia *that an examiner without formal medical education should not perform tests that require manual fixation, with the possible exception of cervical rotation. Abdominal endurance should be tested in the same way as in our study; the Biering-Sørensen test with our modification should not be used; and the modified PILE used in this study could be recommended.

Although the difference was not significant, the proportion of tests with acceptable inter-rater reliability tended to be higher for the patients than for the healthy subjects (seven vs. four tests). That is in line with previous research [19,21]. The intra-rater reliability of the package tended to be greater than the inter-rater reliability, which also corresponds with other studies [19,35]

The study has several limitations, which diminish the generalizability of the results. One weakness was that the gold standard consisted of one single physiotherapist. For example, the active-straight-leg-raise and cervical bending showed an acceptable intra-reliability for both the physiotherapist and the research assistant, while the inter-reliability for those tests was poor (see Table 1 and 2). The reason for that could, hypothetically, be that the research assistant, not the physiotherapist, performed those tests more reliably. However, the substantially narrower 95% CI and lower SEM of the physiotherapist (see Table 2) indicate the opposite. Also, the use of only one examiner without medical education is a limitation. The total lack of previous references concerning the use of examiners without medical education makes it difficult to evaluate the representativeness of the medically untrained examiner of our study. Another weakness was that the intra-reliability study only included a comparatively small number of healthy subjects. A way to overcome the ethical and methodological difficulties of using patients for as many as three examinations is to spread them out over several days, as in the studies of Ljungquist et al. [4] and Horneij et al. [13]. This option, however, was beyond the limits of the resources of our study. The intra-rater reliability study was limited to ten participants for each examiner. Ljungquist et al. [4] used as few as 11 healthy subjects in one of the two samples for studying the intra-rater reliability of an 11-test package. They all performed all the tests on every test occasion, which made a valuable contribution to the comprehensive assessment of the package. In the other sample used by Ljungquist et al., 24 patients with back or neck pain were engaged. Although the examinations were distributed over three different days, only 16 of them performed all 11 tests each time, mainly because of pain. This illustrates the problems involved in engaging patients in numerous examinations.

Notwithstanding its limitations, this study indicates that even an examiner with no formal medical education could be used without loss of quality, at least for tests that require no manual fixation. This might produce a better assessment of outcome at defensible cost and might also be useful in a research context. To make our results more generalizable and their implications more searching, a similar study should be conducted with two or more examiners with and without formal medical education, and the intra-rater reliability study should also include patients and involve more participants.

When the complete data of the randomized controlled trial (see Background) are available, the measurement results of the tests with poor reliability should be interpreted with caution.

## Conclusion

Given a 10-test package for patients with prolonged back and neck pain, an examiner without formal medical education could be used without loss of quality, at least for the five tests that require no manual fixation. This might produce a better assessment of outcome at defensible cost and might also be useful in a research context. To make our results more generalizable and their implications more searching, a similar study should be conducted with two or more examiners with and without formal medical education, and the intra-rater reliability study should also include patients and involve more participants.

## Competing interests

The authors declare that they have no competing interests.

## Authors' contributions

OL and LE participated in the design of the study and coordinated and monitored the performance of the tests and data collection. OL performed the statistical analyses and prepared drafts of the manuscript. LES, as supervisor for OL and LE, participated in all phases of the study. All authors read and approved the final manuscript.

## Pre-publication history

The pre-publication history for this paper can be accessed here:



## Figures and Tables

**Table 1:** Inter-rater reliability. Fifty participants tested by A (the physiotherapist) and B (the research assistant). The five tests that required manual fixation are italicized. ICC in bold text indicates acceptable ICC (> .60). The mean difference between the measurements by A and B is compared, *p*-value in bold text indicates a significant difference (*p *< .05). + indicates acceptable, – indicates poor inter-rater reliability.

10-test package (including 16 sub-tests):	Forward bending (cm)	Modified Schober (cm)	Lateral bending (cm)		*Trunk rotation (°)*		*Active-straight-leg raise (°)*		*Cervical bending (°)*		*Cervical rotation (°)*		Abdom. endurance (seconds)	*Mod. Biering-Sørensen (sec.)*	Modified PILE (kg)	
			Right	Left	*Right*	*Left*	*Right*	*Left*	*Forward*	*Backward*	*Right*	*Left*			Lumbar	Cervical
**All of the 50 participants**																
ICC	**.99**	**.79**	**.93**	**.95**	**.82**	**.85**	**.94**	**.90**	**.61**	**.84**	**.70**	**.69**	**.92**	**.91**	**.97**	**.97**
95% CI of ICC	.98–1.00	.67–.88	.89–.96	.91–.97	.70–.89	.75–.91	.91–.97	.86–.95	.45–.78	.78–.92	.54–.83	.51–.81	.87–.96	.85–.95	.95–.98	.94–.98
SE of measurement	1.2	.7	1.3	1.1	6	6	4	6	7	5	6	6	8	16	2.2	1.8
Mean	6.4	6.8	17.9	18.1	48	47	68	70	52	65	65	68	32	79	27.8	19.3
Mean difference	-.1	.2	.3	.4	1	-1	3	4	4	3	2	1	-2	-8	.5	.4
95% CI of mean diff.	-.6–.4	-.1–.5	-.2–.8	-.1–.9	-1–3.7	-2.8–1.8	1.2–4.6	1.6–6.0	1.2–6.7	1.3–5.1	-.4–4	-1.0–3.9	-5.4–1.4	-14.3–1.1	-.4–1.3	-.3–1.2
*p*-value	NS	NS	NS	NS	NS	NS	**.002**	**.001**	**.006**	**.001**	NS	NS	NS	**.02**	NS	NS
Inter-rater reliability	**+**	**+**	**+**	**+**	**+**	**+**	-	-	-	-	**+**	**+**	+	-	**+**	**+**
**30 patients**																
ICC	**.99**	**.94**	**.98**	**.97**	**.85**	**.88**	**.96**	**.96**	.52	**.81**	**.64**	**.68**	**.90**	**.96**	**.98**	**.98**
95% CI of ICC	.98–1.00	.90–.97	.93–.98	.95–.98	.74–.91	.81–.93	.95–.98	.94–.98	.36–.74	.69–.89	.44–.78	.49–.80	.85–.95	.92–.98	.96–.99	.96–.99
SE of measurement	1.4	.4	1.0	.9	6	5	4	4	8	5	6	7	6	10	2.1	1.5
Mean	9.2	6.6	16.4	16.8	46	43	64	65	48	60	61	66	16	54	24.6	17.2
Mean difference	.0	.2	.1	-.2	1	2	2	2	5	4	2	-1	-3	-2	.3	-.1
95% CI of mean diff.	-.8–.8	-.1–.4	-.5–.6	-.7–.3	-1.6–4.3	-.9–4.3	.1–3.9	.2–4.2	.8–8.9	.9–6.3	-1.7–4.9	-4.1–3.2	-6.0–.2	-7.3–3.5	-.8–1.4	-9–.6
*p*-value	NS	NS	NS	NS	NS	NS	**.04**	**.04**	**.02**	**.01**	NS	NS	**.04**	NS	NS	NS
Inter-rater reliability	**+**	**+**	**+**	**+**	**+**	**+**	-	-	-	-	**+**	**+**	-	+	**+**	**+**
**20 healthy subjects**																
ICC	**.95**	.22	**.79**	**.85**	**.75**	**.75**	**.84**	**.70**	.59	**.86**	**.66**	**.63**	**.86**	**.69**	**.95**	**.94**
95% CI of ICC	.92–.97	.07–.46	.68–.89	.84–.95	.59–.85	.64–.87	.78–.92	.62–.86	.40–.76	.80–.93	.49–.80	.58–.84	.76–.92	.59–.85	.92–.97	.91–.97
SE of measurement	.9	1.0	1.5	1.1	6	6	5	7	6	4	5	4	12	22	2.3	2.1
Mean	2.2	7.1	20.1	20.2	50	52	75	77	58	72	70	72	55	116	32.5	22.4
Mean difference	-.3	.3	.8	1.4	2	-4	4	6	3	3	2	4	0	-16	.7	1.3
95% CI of mean diff.	-.8–.3	-.4–.9	-.3–1.8	.6–2.1	-2.4–5.4	-7.8–.3	.8–7.6	1.5–10.8	-1.2–6.3	.0–5.3	-1.0–5.2	1.6–7.0	-8.0–7.3	-30.7–2.1	-.8–2.2	-.1–2.7
*p*-value	NS	NS	NS	**.001**	NS	NS	**.02**	**.01**	NS	**.047**	NS	**.004**	NS	**.03**	NS	NS
Inter-rater reliability	+	-	+	-	+	+	-	-	-	-	+	-	+	-	**+**	**+**

ICC = Intra-class-correlation coefficient. NS = Not significant. SE = Standard error

**Table 2:** Intra-rater reliability. Twenty healthy subjects tested twice by A or B. Further explanations in Table 1.

10-test package (including 16 sub-tests):	Forward bending (cm)	Modified Schober (cm)	Lateral bending (cm)		*Trunk rotation (°)*		*Active-straight-leg raise (°)*		*Cervical bending (°)*		*Cervical rotation (°)*		Abdom. endurance (sec)	*Mod. Biering-Sørensen (sec.)*	Modified PILE (kg)	
			Right	Left	*Right*	*Left*	*Right*	*Left*	*Forward*	*Backward*	*Right*	*Left*			Lumbar	Cervial
**Examiner A**																
ICC	**.95**	**.87**	**.99**	**.94**	**.92**	**.96**	**.99**	**.97**	**.86**	**.98**	**.94**	**.86**	**.90**	**.92**	**.93**	**.95**
95% CI of ICC	.89–.99	.68–.96	.95–1.00	.82–.98	.76–.97	.87–.99	.96–1.00	.92–.99	.67–.96	.95–.99	.82–.98	.63–.95	.75–.97	.75–.97	.80–.98	.86–.98
SE of measurement	.9	.3	.5	1.0	3	3	2	3	2	2	2	3	9	16	2.3	1.5
Mean	2.5	7.1	21.2	21.0	55	53	75	78	58	75	72	74	66	117	31.8	20.8
Mean difference	-.7	.2	-.1	.1	1	-1	0	1	2	1	1	-1	7	4	.8	.4
95% CI of mean diff.	-1.6–.1	-.1–.5	-.5–.5	-.9–1.1	-2.6–3.6	-3.8–1.8	-1.6–1.4	-1.7–3.3	-.8–4.0	-.5–2.5	-1.4–3.0	-3.8–1.4	-2.7–15.7	-12.3–19.3	-1.6–3.2	-1.1–1.9
*p*-value	NS	NS	NS	NS	NS	NS	NS	NS	NS	NS	NS	NS	NS	NS	NS	NS
Intra-rater reliability	**+**	**+**	**+**	**+**	**+**	**+**	**+**	**+**	**+**	**+**	**+**	**+**	+	+	**+**	**+**
**Examiner B**																
ICC	**.95**	**.79**	**.73**	**.86**	.46	**.83**	**.97**	**.93**	**.62**	**.80**	**.82**	**.82**	**.65**	.20	**.97**	**.94**
95% CI of ICC	.86–.98	.46–.93	.37–.91	.61–.95	.13–.85	.54–.94	.90–.99	.78–.97	.12–.85	.53–.94	.53–.94	.52–.94	.18–.86	.14–.85	.89–.99	.83–.98
SE of measurement	.9	.7	1.6	1.4	7	5	3	4	6	5	4	4	17	17	2.4	2.9
Mean	1.8	7.2	19.8	19.7	48	51	70	76	57	67	68	67	46	104	32.8	23.5
Mean difference	.4	.2	-.7	-.2	-8	-1	0	-1	-1	3	-1	-1	3	33	.0	-1.8
95% CI of mean diff.	-.5–1.3	-.5–.9	-2.3–.9	-1.6–1.2	-14.7–.3	-6.3–4.3	-2.8–3.0	-4.9–3.5	-6.7–5.6	-2.0–7.6	-5.4–2.6	-4.7–2.7	-14.3–20.5	15.3–50.5	-2.4–2.4	-4.7–1.1
*p*-value	NS	NS	NS	NS	**.04**	NS	NS	NS	NS	NS	NS	NS	NS	**.002**	NS	NS
Intra-rater reliability	+	+	+	+	-	+	+	+	+	+	+	+	+	-	+	+
